# A Novel Arginine to Tryptophan (R144W) Mutation in Troponin T (*cTnT*) Gene in an Indian Multigenerational Family with Dilated Cardiomyopathy (FDCM)

**DOI:** 10.1371/journal.pone.0101451

**Published:** 2014-07-03

**Authors:** Deepa Selvi Rani, Perundurai S. Dhandapany, Pratibha Nallari, Calambur Narasimhan, Kumarasamy Thangaraj

**Affiliations:** 1 CSIR-Centre for Cellular and Molecular Biology, Hyderabad, Telangana, India; 2 Department of Genetics and Genomic Sciences, Icahn School of Medicine at Mount Sinai, New York, New York, United States of America; 3 Department of Genetics, Osmania University, Hyderabad, Telangana, India; 4 Department of Cardiology, CARE Hospitals, Hyderabad, Telangana, India; University of Hyderabad, India

## Abstract

Cardiomyopathy is a major cause of heart failure and sudden cardiac death; several mutations in sarcomeric protein genes have been associated with this disease. Our aim in the present study is to investigate the genetic variations in Troponin T (*cTnT*) gene and its association with dilated cardiomyopathy (DCM) in south-Indian patients. Analyses of all the exons and exon-intron boundaries of *cTnT* in 147 DCM and in 207 healthy controls had revealed a total of 15 SNPs and a 5 bp INDEL; of which, polymorphic SNPs were compared with the HapMap population data. Interestingly, a novel R144W mutation, that substitutes polar-neutral tryptophan for a highly conserved basic arginine in *cTnT*, altering the charge drastically, was identified in a DCM, with a family history of sudden-cardiac death (SCD). This mutation was found within the tropomyosin (*TPM1*) binding domain, and was evolutionarily conserved across species, therefore it is expected to have a significant impact on the structure and function of the protein. Family studies had revealed that the R144W is co-segregating with disease in the family as an autosomal dominant trait, but it was completely absent in 207 healthy controls and in 162 previously studied HCM patients. Further screening of the proband and three of his family members (positive for R144W mutant) with eight other genes *β-MYH7, MYBPC3, TPM1, TNNI3, TTN, ACTC, MYL2* and *MYL3*, did not reveal any disease causing mutation, proposing the absence of compound heterozygosity. Therefore, we strongly suggest that the novel R144W unique/private mutant identified in this study is associated with FDCM. This is furthermore signifying the unique genetic architecture of Indian population.

## Introduction

Dilated cardiomyopathy (DCM: OMIM 115200), is characterized by cardiac left ventricular dilation and systolic dysfunction, affects at least 1 in 2500 individuals [1], and a major cause for morbidity and mortality [2], including heart failure (HF) and sudden cardiac death (SCD) [3]–[5]. Familial DCM (FDCM) is a genetically heterogeneous disease [6], whereas Idiopathic DCM (IDCM) is diagnosed when clinically detectable causes of DCM are excluded. Genetic screening of first-degree relatives had revealed, approximately 20 to 35% of idiopathic cases, were due to genetic defects [6]–[11]. More than 30 nuclear genes, encoding for sarcomere (contractile apparatus), cytoskeletal and calcium homeostasis proteins of diverse functions, have been reported to cause FDCM [6]. To date, mutations in *LMNA, MYH7, MYBPC3, TNNT2, SCN5A*, and *MYH6* genes have been accounted for approximately 75% of FDCM [12]. Most of the genes implicated in genetics of DCM/FDCM follow autosomal dominant mode of inheritance [6], though a few follow autosomal recessive, X-linked [10], [13]–[16] and mitochondrial [16], [17]. Recent studies had suggested that the double and triple mutations identified in sarcomere protein genes were found to be associated with early onset of HCM [18], [19].

Indian populations are reported to be more prone to cardiac disorders, which might be due to their high effective population size (Ne) and lifestyle, resulting a unique genetic structure [20]–[22]. Our previous study on cardiac Troponin I3 (*TNNI3*) [23], [24] and Troponin T2 (*TNNT2*) [25] in hypertrophic cardiomyopathy (HCM), and cardiac actin (*ACTC*) [26], myosin binding protein C (*MyBPC3*) [20], had revealed few variants, of which a 25 bp deletion was found to be associated with both HCM and DCM in India and south Asia [20]. Unfortunately, not many studies have been conducted on Indian patients to explore the genetic etiology of the disease, particularly with reference to the sarcomere protein genes. Our aim in the present study is to investigate the genetic variations in Troponin T (*cTnT*) gene, and its association with DCM in South Indian cohorts.

## Results

**Figure 1:**
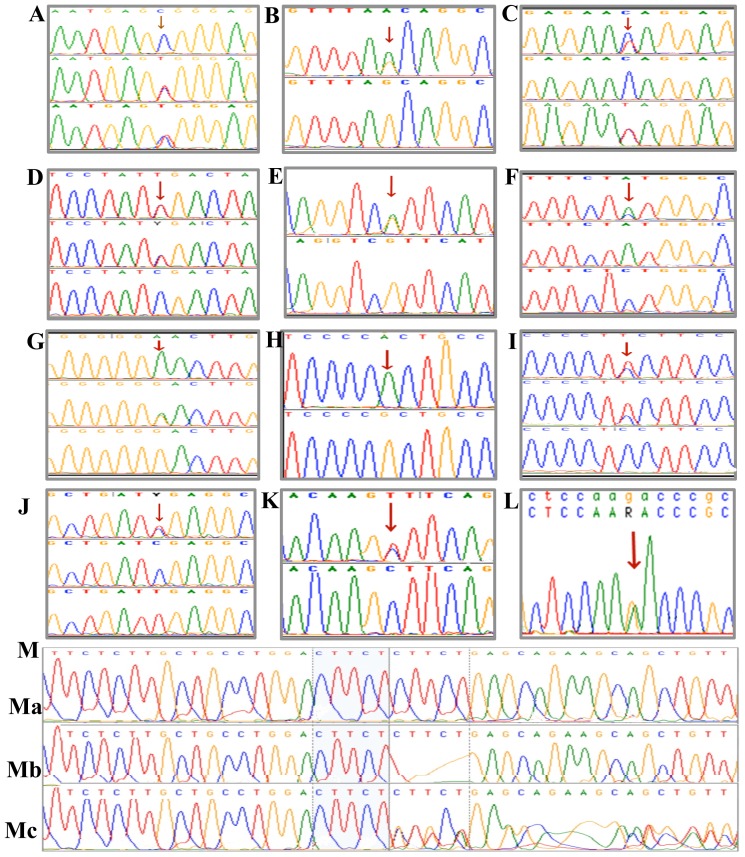
1A-1M: Electropherograms showing SNPs of cTnT gene, observed in the present study on South Indian dilated cardiomyopathy patients. Mutation sites were shown with arrows. **Fig. 1A** **. R144W** [rs483352832]: Electropherogram (arrow) showing a novel missense mutation (R144W) at the nucleotide position g.14351 of human cTnT gene. The upper lane showing sequences of homozygous wild type allele ‘C’ in a control individual. The middle and the lower lanes were showing the sequences of heterozygous (C/T  = Y) alleles in two individuals (a DCM patient and his relative, respectively). **Fig. 1B. G>A [IVS11-1G]** [rs483352835]: Electropherogram (arrow) showing a variant at splice acceptor site of human *cTnT* gene at nucleotide position g.16283, the electropherogram of a upper lane showing sequence of heterozygous (A/G = R) variant in a DCM patient, the lower lane showing sequence of control individual having wild type allele ‘G’ (homozygous). **Fig. 1C. N164N** [rs483352833]: Electropherogram (arrow) showing a novel synonumous mutation (N164) at the nucleotide position g.15304 of human *cTnT* gene in 2 DCM patients. The upper lane shows the sequences of heterozygous (C/T  = Y) transition in a DCM patient. The middle lane was the sequences of a control individual showing the wild type allele ‘C’ (homozygous). The lower lane sequences showing heterozygous (C/T  = Y) transition was from a 2^nd^ DCM patient. **Fig. 1D**. [rs3729842]: Electropherogram showing (arrow) a single nucleotide polymorphism at the nucleotide position g.10636 (C/T = Y) in intron 5 of human *cTnT* gene. The upper and the middle lanes were sequences showing heterozygous (C/T = Y) transition in DCM patients, the lower lane showing homozygous wild type (C/C) allele in a control individual. **Fig. 1E**. [rs3729845]: Electropherogram showing (arrow) at the nucleotide position g.13011 of human *cTnT* gene. The upper lane showing sequences of the heterozygous (A/G  = R) transition, and the lower lane showing homozygous wild type (G/G) allele of a control. **Fig. 1F**. [rs1104859]: Electropherogram showing (arrow) at the nucleotide position **g.11643** (A/C = M) in Intron 11 of human *cTnT* gene. The upper lane sequences showing the heterozygous (A/C = M) transversion, the middle lane showing homozygous wild type (G/G), and the lower lane sequences showing mutant homozygous (C/C) allele. **Fig.1G** **. SNP-rs3729843**: Electropherogram showing (arrow) a SNP at the nucleotide position g.10822 (G/A = R) in intron 5 of human *cTnT* gene. The upper lane sequences showing mutant homozygous (A/A) allele. The middle lane sequences showing heterozygous (G/A = R) transition allele, and the lower lane showing sequences of homozygous wild type (G/G) allele in a control individual. **Fig. 1H**. [rs45576939]: Electropherogram showing (arrow) a novel mutation G>A at nucleotide position g.10370 in intron 4 of human *cTnT* gene, the upper lane displaying homozygous mutant (A/A) allele, and the lower lane showing sequences of a wild type allele (G/G). **Fig. 1I**. [rs45576635]: Electropherogram showing (arrow) a SNP at the nucleotide position g.14492 (C/T = Y) in intron 15 of human *cTnT* gene, the upper and the middle lanes sequences displaying heterozygous (C/T = Y) transition, and the lower lane sequences showing homozygous wild type (C/C) allele. **Fig. 1J**. [rs3729547]: Electropherogram showing (arrow) a polymorphic variant at the nucleotide position g.13424 of human *cTnT* gene, the upper lane displaying sequences of the heterozygous (C/T  = Y) transition, the middle lane sequences showing homozygous wild type (C/C) allele, and the lower lane displaying sequences of the homozygous mutant (T/T) allele. **Fig. 1K**. [rs483352834]. Electropherogram (arrow) showing a novel mutation at the nucleotide position g.15179 C>T in intron 11 of human *cTnT* gene, the upper lane displaying sequences of a DCM patient having heterozygous (C/T) transition, and the lower lane exhibiting sequences of a control individual having homozygous wild type allele (C/C). **Fig. 1L. K276K**. [rs483352836]: Electropherogram (arrow) exhibiting novel synonumous (K276) variant at the nucleotide position g.19429 of human *cTnT* gene in a DCM patient, the DCM patient displaying heterozygous (G/A  = R) transition. **Fig. 1M**. Sequence electropherogram showing (CTTCT) 5 bp Polymorphism. **Ma**. Presence of two copies of CTTCT (Insertion/Insertion – homozygous insertion) in both the chromosomes, **Mb**. Absence of one copy of CTTCT (Deletion/Deletion – homozygous deletion in both the chromosomes, **Mc**. Presence of 2 copies of CTTCT in one chromosome and presence of one copy of CTTCT in another chromosome (Insertion/deletion – heterozygous allele). g.6626-30 (5 bp).

Sequencing of all the exons and the exon-intron boundaries (5373 bp) of Troponin T2 (*cTnT*) gene in 147 DCM patients along with 207 healthy controls had revealed a total of 15 SNPs and a 5 bp INDEL (Fig. 1A to 1M and Table 1).

**Table 1:** Total number of mutations observed in Troponin T (*cTnT*) gene.

S:NO	Chromosome position	Genomic position	Major >Minor allele	Location	SNP Reference	AA Change	Novel	PolyPhen_2	SIFT	Predictions	CON/207	DCM/147
1	1201341276-80	g.6626-30	[5 bp]	Intron 3	5 bp	_	_	_	_	_	HP	HP
2	1201337436	g.10370	G>A	Intron 4	rs45576939	_	_	_	_	_	0	1
3	1201337170	g.10636	C>T	Intron 5	rs3729842	_	_	_	_	_	HP	HP
4	1201336984	g.10822	G>A	Intron 6	rs3729843	_	_	_	_	_	HP	HP
5	1201335899	g.11907	A>G	Intron 7	rs1573230	_	_	_	_	_	1	0
6	1201334795	g.13011	G>A	Exon 8	rs3729845	S69S	_	_	_	_	0	2
7	1201334382	g.13424	C>T	Exon 9	rs3729547	I106I	_	_	_	_	HP	HP
8	1201333455	g.14351	C>T	Exon 10	rs483352832	R144W	Novel	Damaging	Damaging	Pathogenic	0	1
9	1201332502	g.15304	C>T	Exon 11	rs483352833	N164N	Novel	_	_	_	0	2
10	1201332603	g.15179	C>T	Intron 11	rs483352834	Novel	Novel	_	_	_	0	1
11	1201331554	g.16252	[AC]	Intron 11	rs1104859	_	_	_	_	_	HP	HP
12	1201331523	g.16283	G>A	Intron 11	rs483352835	SS	_	_	_	_	0	1
13	1201328824	g.18982	C>T	Intron 14	rs2275863	_	_	_	_	_	P	P
14	1201328705	g.19101	C>T	Intron 15	rs45576635	_	_	_	_	_	0	2
15	1201328913	g.18893	C>T	Intron 16	rs45509695	_	_	_	_	_	0	4
16	1201328377	g.19429	G>A	Exon 16	rs483352836	K276K	_	_	_	_	0	2

*SNP- single nucleotide polymorphism, AA-Amino Acid, CON- Controls, DCM- Dilated cardiomyopathy, SS- Splice Site, HP-Highly Polymorphic.

### Arginine to Tryptophan substitution at residue 144 (R144W) of cTnT gene

**Figure 2:**
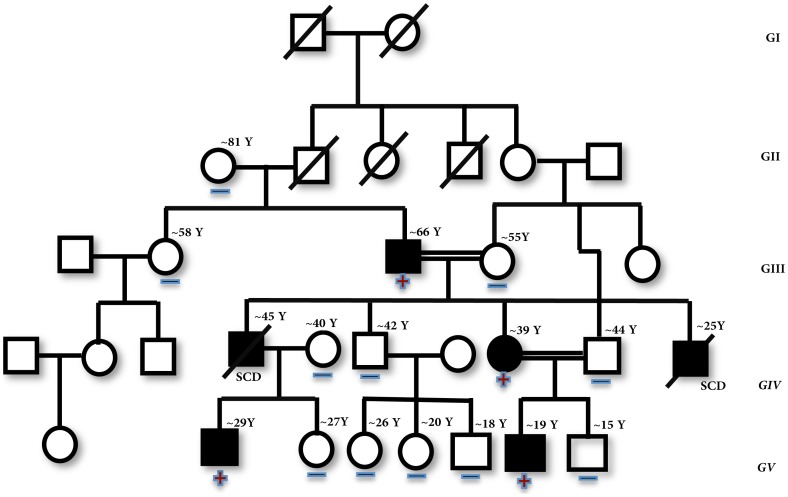
The pedigree of a familial dilated cardiomyopathy patient (FDCM) having R144W mutation in the exon 10 of cardiac Troponin T2 (*cTnT*) gene. Squares indicate males; circles, females; open symbols, normal individuals; solid symbols, affected individuals, Slanted bars indicate deceased members of family. Plus signs indicate the presence of R144W mutation in *cTnT*; minus signs suggest the absence of mutation R144W in *cTnT*.

**Figure 3:**
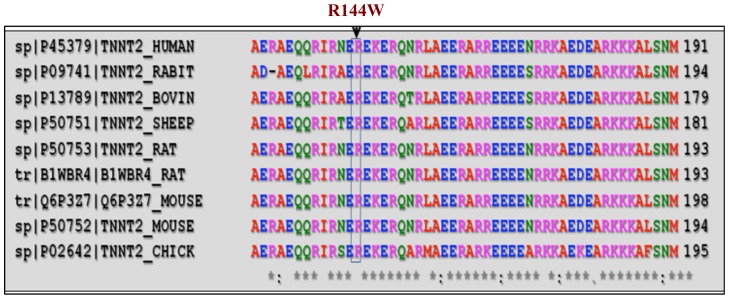
The amino acid arginine at residue 144 in human Troponin T (*cTnT*) is highly conserved across many species, including mouse, rat, chicken, rabbit, sheep and bovine.

Of the 15 SNPs, a unique c.430 C>T transition (GenBank No. NM_000364) in exon 10 of *TNNT2* gene, identified in a 29 years old male DCM patient, is of great interest, as the mutation replaces the highly conserved basic amino acid arginine at residue 144 to polar-neutral tryptophan R144W [rs483352832] (Fig. 1A). The R144W mutation has resulted with loss of restriction sites; *Mbil* 19, *Acil* 19, *BsrBl* 19, *AccBSl* 19. Subsequent, screening of this (R144W) mutation with available family members had revealed its presence in three other individuals with DCM phenotype (Fig. 2). However, this mutation was absent in 207 healthy unrelated controls, and in 162 HCM patients [25]. Multiple alignment of the amino acid with different species had revealed that the arginine at 144 in human *cTnT* is evolutionarily conserved across species; including mammals, birds, reptiles, and nematode (Fig. 3).

While interacting with the family members of the proband, a history of sudden cardiac deaths (SCD) in the family was noted. Two individuals in the family, who were diagnosed with DCM, had died due to severe congestive heart failure at the age of 45 and 25 years. However, a 66-year-old individual in the same family with DCM having mild symptoms have also been noticed. Thus indicating that the age of onset, and the severity of the disease are highly variable within the family (Fig. 2 and, Table 2).

**Table 2:** Clinical details of the family members carrying R144W mutation.

Generations (G)	Sex	Genotype (R144W) Positive	Age (in years)	Age of onset (in years)	NYHA III or IV	Mitral Regurgitation	Ventricular arrhythmia	LVIDd (mm)	LVEF (%)
G-III^rd^	Male	YES	66	60	YES	MOD	YES	67	30
G-IV^th^	Male	NK (SCD)	45	44	YES	SEV	YES	NK	NK
G-IV^th^	Female	YES	39	30	YES	MOD	YES	71	29
G-IV^th^	Male	NK (SCD)	25	24	YES	SEV	YES	NK	NK
G-V^th^	Male	YES	29	25	YES	SEV	YES	72	26
G-V^th^	Male	YES	19	15	YES	MILD	NO	55	34

SCD- Sudden cardiac death; NYHA-New York Heart Association; LVIDd- left ventricular internal diastolic dimension; LVEF- left ventricular ejection fraction.

### A novel splice acceptor site variant

We have also identified a novel splice acceptor site variant (G→A) in intron 12 of *cTnT* gene [rs483352835], in a 63 years old male DCM patient (Fig. 1B and, Table 1). This patient had both dilated LV/LA, with EF 25%, global hypokinesia, grade III systolic dysfunction, and IVS thinned out 7 mm. Unfortunately, we were unable to get the family samples for additional analyses.

### Two novel synonymous mutations

We further identified two novel synonymous mutations, N164N (C→T; [rs483352833]) and K276K (G→A; [rs483352836]) in *cTnT* gene (Fig. 1C and 1L, Table 1) exclusively in DCM. Of which, N164N (Fig. 1C) was observed in 2 DCM (2/147 = 1.4%) patients with EF of 35% (a 35 year old female) and 30% (39 year old male). The codon bias analysis had revealed a replacement of more frequently used (wild type) codon (AAC: 64%) with a less frequent one (AAT: 36%) (Table 3). The female patient showed both dilated left ventricle and atrium, moderate mitral regurgitation and moderate LV systolic dysfunction, while the male patient showed LV dilation and moderate LV systolic dysfunction.

**Table 3:** The codon usage in human cTnT (Genbank NO. NM_000364) gene.

S:NO	Chr. position	Position	SNP. Ref	Location	Nt. Change	A.A Site	Type	Codon	Amino acid	Fraction	%	Frequency	Codon Useage
1	1201334795	g.13011	rs3729845	Exon 8	G/A	79^th^	Wild	TCG	Serine (S)	0.125	13	3.46	1
							Mutant	TCA	Serine (S)	0	0	0	0
2	1201334382	g.13424	rs3729547	Exon 9	C/T	116^th^	Wild	ATC	Isoleucine (I)	0.667	67	27.68	8
							Mutant	ATT	Isoleucine (I)	0.25	25	10.381	3
3	1201332502	g.15304	**rs483352833**	Exon 11	C/T	164^th^	Wild	AAC	Asparagine (N)	0.692	69	31.142	9
							Mutant	AAT	Asparagine (N)	0.308	31	13.841	4
4	1201328377	g.19429	**rs483352836**	Exon 16	G/A	276^rd^	Wild	AAG	Lysine (K)	0.758	76	86.505	25
							Mutant	AAA	Lysine (K)	0.242	24	27.682	10

Chr- Chromosome, SNP- Single Nucleotide Polymorphism, Ref- References, Nt. Nucleotide, A.A-Amino Acid.

The K276K synonymous mutation (Fig. 1L; rs483352836) was observed in 2 DCM patients (2/147 = 1.4%), which replaces very frequent codon (71%; AAG) with the less frequent codon (29%; AAA) (Table 3). Though these two (N164N; K276K) mutations were synonymous, its exclusive presence in dilated cardiomyopathy patients, illustrates its possible role in disease pathogenesis, however, they need to be studied further.

### Two intronic SNPs and their splicing patterns

We found two intronic SNPs of *cTnT* gene (G→A; g.10370_ [rs45576939] and C→T; g.15179- [rs483352834]), exclusively in DCM patients. *In silico* analyses had predicted abnormal splicing pattern (Table 4). The G→A variant was found to create an additional binding site for hnRNP. K1K2 (Fig. 1H and, Table 4), while the C→ T variant was also causes drastic changes by altering a total of 4 binding sites, 2 each in hnRNPs and SR proteins (SRP20 ASF/SF2, SC35 and U2AF65) (Fig. 1K and, Table 4), indicating its regulatory role, however, its clinical significance need to be studied further.

**Table 4:** The hn-RNP's and SR-proteins binding site sequences in controls and DCM as predicted by “Splicing Rainbow” tool.

S. No	Chromosome Position/rs number	Position	Location	Splicing Rainbow & the binding site sequences	
				Normal	Mutant
1	1201337436/rs45576939	g.10370(G>A)	Intron 4	**hnRNP.K1K2**	
				CCCCATCCCCA	CCCCATCCCCA
				GCCCAT	GCCCAT
				_	TCCCCA
2	1201332603/rs483352834	g.15179 (C>T)	Intron 10	**SRP20**	
				AGCTTCAGC	_
				**ASF/SF2**	
				CTGAACTCACCCATAAAGACC	CTGAACTCACCCATAAAGACCACAAGT
				**C35S**	
				GACCCAAGCTTCAG	GACCACAA
				**U2AF65**	
				_	TTTC

### Polymorphic SNPs

The chi-square and fisher exact probability test was done to test the significance of polymorphic SNPs that were observed in this study (Table 5). We have compared the genotype and allele frequencies of these SNPs (NCBI database; www.ncbi.nlm.nih.gov/projects/SNP/snp), with HapMap population's data, (HER_ASIAN-PANEL; HER_HISP-PANEL; HER_CEPH-PANEL; HER_YORUB-PANE).

**Table 5:** Chi-square and Fisher Exact Probability Test for SNP's found in this study.

SNPs	Alleles	Controls (%)	DCM (%)	Odds Ratio	0.95 Confidence Intervals (Observed)		Chi-square		Fisher Exact Probability Test	
					Lower Limit	Upper Limit	Yates	Pearson	P (one-tailed)	P (two-tailed)
5 bp pol	Deletion allele	56.5	69	1.679	0.94	2.99	0.107	0.079	0.0534	0.107
	Insertion allele	43.5	31							
rs3729842	Major allele	95	90	0.474	0.156	1.411	0.28	0.18	0.14	0.282
	Minor allele	5	10							
rs3729843	Major allele	62	57	0.813	0.462	1.431	0.57	0.471	0.28	0.565
	Minor allele	38	43							
rs3729547	Major allele	80	81	1.066	0.529	2.146	1	0.862	0.5	1
	Minor allele	20	19							
rs1104859	Major allele	71	78	1.448	0.763	2.748	0.33	0.256	0.17	0.33
	Minor allele	29	22							
rs2275863	Major allele	76	81	1.346	0.683	2.653	0.49	0.389	0.246	0.491
	Minor allele	24	19							
rs3729845	Major allele	100	98	-	-	-	-	-	0.249	0.497
	Minor allele	0	2							

*SNP- single nucleotide polymorphism, DCM- Dilated cardiomyopathy.

**Figure 4:**
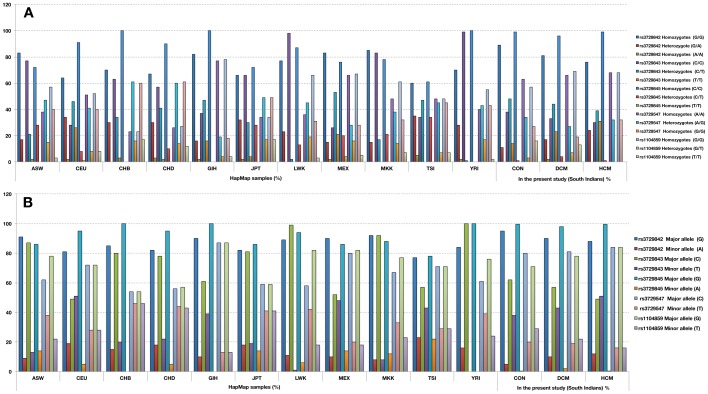
A. The genotype frequencies of SNPs (rs3729842, ra3729843, rs3729845, rs3729547, rs1104859) in the present study were compared with HapMap samples (various populations). **B**. The Allele frequencies of SNPs (rs3729842, rs3729843, rs3729845, rs3729547, rs1104859) in the present study were compared with HapMap samples (various populations). HapMap samples (various populations)- ASW, African Ancestry in SW USA; CEU, CEPH Collection; CHB, Han Chinese in Beijing, China; CHD, Chinese in Metropolitan Denver, CO; GIH, Gujarati Indians in Houston, TX; JPT, Japanese in Tokyo, Japan; LWK, Luhya in Webuye, Kenya; MEX, Mexican Ancestry in LA,CA; MKK, Maasai in Kinyawa, Kenya; TSI, Toscani in Italia; YRI, Yoruba in Ibadan, Nigeria; CON-controls; HCM-hypertrophic cardiomyopathy; DCM-dilated cardiomyopathy.

*a) SNP*-rs3729842: The homozygous mutant allele was exclusively observed in DCM and completely absent in the normal controls and HapMap populations (ASW, CHB, LWK, MKK) (Fig. 1D and, Fig. 4A and 4B). *b) SNP*-rs3729843: The allele frequencies of DCM have matched only with MXL, TSI, HapMap populations. The minor allele frequency was low in CEU population, while it was completely absent in two (LWK and YRI) HapMap populations (Fig. 1G and, Fig. 4A and 4B). *c) SNP*-rs3729845: About 4%of heterozygous genotype was observed in DCM, but it was completely absent in the controls, and two (CHB, JPT) of the HapMap populations (Fig. 1E and, Fig. 4A and 4B). *d) SNP*-rs3729547: The frequency of mutant homozygous allele was 7% in DCM as seen in Gujarati Indians GIH (Hap-map sample), but it was as low as 3% in controls (Fig. 1J and, Fig. 4A and 4B). *e) SNP*-rs1104859: The percentage of homozygous mutant allele was 13% in DCM, it was very low (6%) in controls. The frequency of the heterozygous genotype was found to be high in CHB, CHD, JPT, HapMap population's (Fig. 1F and, Fig. 4 A and B).

### Linkage disequilibrium

**Figure 5:**
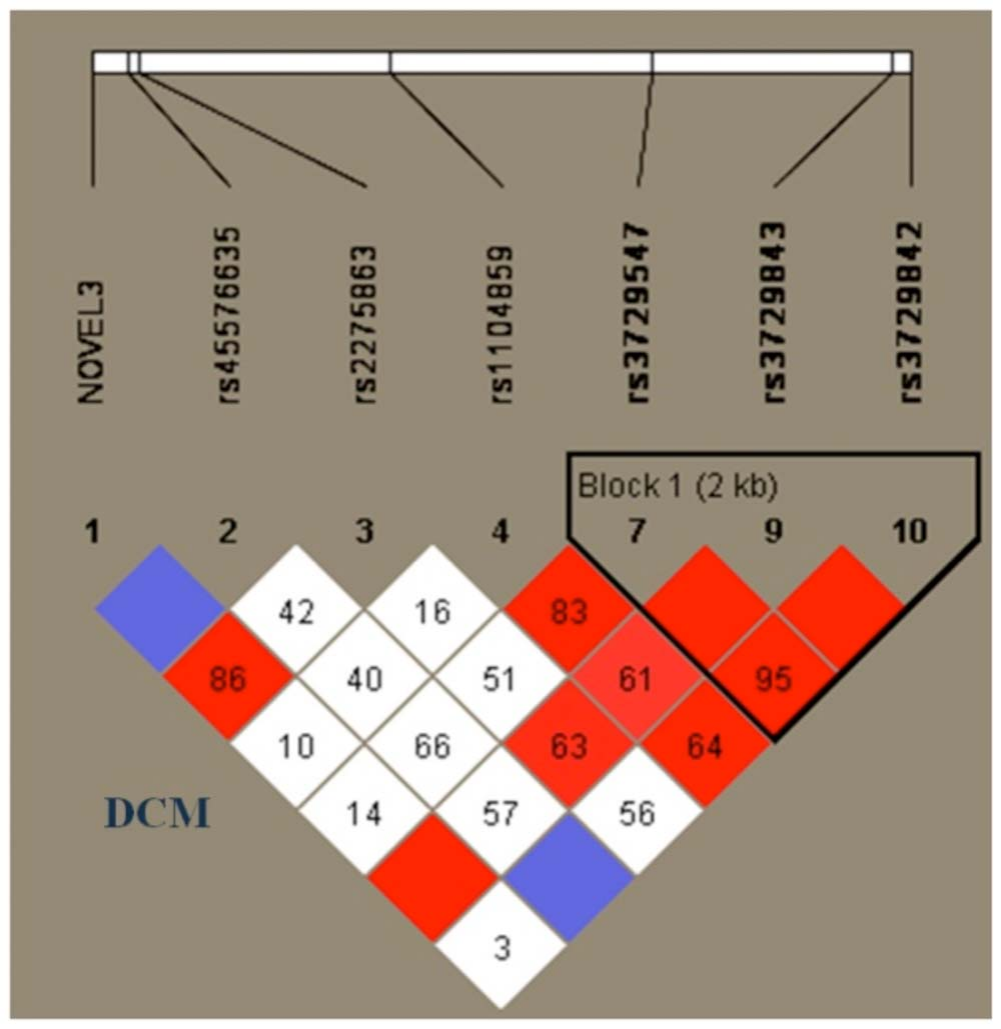
Three SNPs, [rs3729547 (C/T), rs3729843 (G/A), rs3729842 (C/T)], (Table.1; Fig.1D, 1G, 1J) in TNNT2 gene observed in the present study, which were about 2.0 kb apart had shown high Linkage disequilibrium (LD). The bright red color indicates very strong LD (LOD = 2D′ = 1), white color no LD (LOD<2D′<1), and pink (LOD = 2D′<1) and blue (LOD<2D′ = 1) indicate intermediate LD (the standard color scheme is used to display LD). The values in the LD blocks show the r2 values in percentages or multiplied by 100.

Plotting of all the SNPs observed in the present study had revealed a strong linkage disequilibrium among three SNPs; rs3729547 (C/T), rs3729843 (G/A), rs3729842 (C/T), (Fig. 1J, 1G and, 1D and Table 1), respectively, which were about 2.0 kb apart, in both HCM [21] and DCM (Fig. 5).

### A 5 bp INDEL (CTTCT) polymorphism

**Figure 6:**
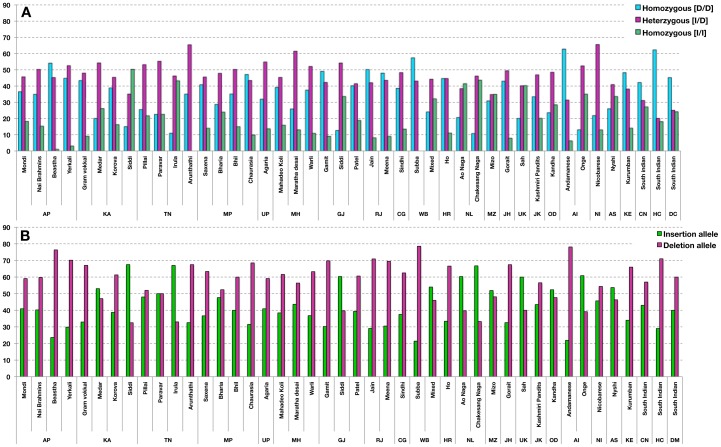
A. The genotype frequency of 5(*cTnT*) gene of DCM, HCM and controls in the present study were compared with the randomly selected individuals from 19 states of India. **B**. The Allele frequency of 5 bp polymorphism observed in Troponin T (*cTnT*) gene of DCM, HCM and controls in the present study were compared with the randomly selected individuals from 19 states of India. Individuals from Rajasthan showed high frequency of Deletion allele, whereas the individuals of northeastern states and HER-YORUB-PANEL of Hap Map population showed high frequency of Insertion allele. AP, Andhra Pradesh; KA, Karnataka; TN, Tamil Nadu; MP, Madhya Pradesh; UP, Uttar Pradesh; MH, Maharashtra; GJ, Gujarat; RJ, Rajasthan; CG, Chhattisgarh; WB, West Bengal; HR, Haryana; NL, Nagaland; MZ, Mizoram; JH, Jharkhand; UK, Uttaranchal; JK, Jammu & Kashmir; OD, Orissa; AI, Andaman Islands; NI, Nicobar Islands; AS, Arunachal Pradesh; KE, Kerala; CN, normal controls; HC, Hypertrophic Cardiomyopathy; DC, Dilated Cardiomyopathy.

A 5 bp (*CTTCT*) polymorphism (Fig. 1M;a-c) that results in skipping of exon 4 of *TNNT2* during splicing was not significant, when compared to normal controls, it was found to be almost equal in DCM however the deletion frequency was high in HCM [25]. We have also further compared the 5 bp (CTTCT) polymorphic frequencies in 2092 randomly selected individuals belonging to 39 ethnic and endogamous populations from 19 states of India (Table 6), with DCM and HCM [25] (Fig. 6 A and B).

**Table 6:** Details of 2092 random population samples from India used to study the 5 bp Deletion Polymorphism.

S. No	States of India	Total Number (Each state)	Name of Tribes	No of Tribes	Genotype Frequency						Allele Frequency		Linguistic Family
					I/D		D/D		I/I		Deletion	Insertion	
					N	%	N	%	N	%	%	%	
1	Andhra Pradesh (AP)	246	Mondi	44	20	45.45	16	36.36	8	18.18	59.085	40.9	Dravidian
			Nai brahmins	46	23	50	16	34.78	7	15.21	59.78	40.21	
			Beastha	91	41	45.05	49	53.84	1	1.09	76.36	23.61	
			Yerkali	65	34	52.3	29	44.61	2	3.07	48.65	29.22	
2	Karnataka (KA)	145	Gram vokkal	44	21	47.72	19	43.18	4	9.09	67.04	32.95	Dravidian
			Medar	50	27	54	10	20	13	26	47	53	
			Korova	31	14	45.16	12	38.7	5	16.12	61.28	38.7	
			Siddi	20	7	35	3	15	10	50	32.5	67.5	
3	Tamil Nadu (TN)	261	Pillai	102	54	52.94	26	25.49	22	21.35	51.96	47.82	Dravidian
			Paravar	40	22	55	9	22.5	9	22.5	50	50	
			Arunthathi	83	54	65.06	29	34.93	0	0	67.46	32.53	
			Irula	36	16	45	4	10.81	16	43.24	33.78	66.75	
4	Madhya Pradesh (MP)	249	Saxena	86	39	45.34	35	40.69	12	13.95	63.36	36.62	Indo-European
			Bharia	42	20	47.61	12	28.57	10	23.8	52.37	47.605	
			Bhil	40	20	50	14	35	6	15	60	40	
			Chaurasia	81	35	43.2	38	46.91	8	9.87	68.51	31.47	
5	Uttar Pradesh (UP)	44	Agaria	44	24	54.54	14	31.81	6	13.63	59.08	40.9	Indo-European
6	Maharashtra (MH)	227	Mahadeo Koli	82	37	45.12	32	39.02	13	15.85	61.58	38.41	Indo-European
			Maratha desai	62	38	61.2	16	25.8	8	12.9	56.4	43.5	
			Warli	83	43	51.8	31	37.34	9	10.84	63.24	36.74	
7	Gujarat (GJ)	231	Gamit	88	37	42.04	43	48.86	8	9.09	69.88	30.11	Proto-Australoid
			Siddi	63	34	53.96	8	12.69	21	33.33	39.67	60.31	Indo-European
			Patel	80	33	41.25	32	40	15	18.75	60.625	39.375	
8	Rajasthan (RJ)	153	Jain	86	36	41.86	43	50	7	8.13	70.93	29.07	Indo-European
			Meena	67	29	38.8	32	47.76	6	8.95	70.1	29.9	
9	Chhattisgarh (CG)	52	Sindhi	52	25	48.07	20	38.46	7	13.46	62.49	37.49	Indo-European
10	West Bengal (WB)	60	Subba	14	6	42.85	8	57.14	0	0	78.565	21.425	Tibeto-Burman
			Mixed	46	11	44	6	24	8	32	46	54	Indo-European
11	Haryana (HR)	9	Ho	9	4	44.44	4	44.44	1	11.1	66.66	33.32	Austro-Asiatic
12	Nagaland (NL)	71	Ao Naga	34	13	38.23	7	20.58	14	41.17	39.695	60.285	Tibeto-Burman
			Chakesang Naga	37	17	45.94	4	10.81	16	43.24	33.78	66.75	Tibeto-Burman
13	Mizoram (MZ)	26	Mizo	26	9	34.61	8	30.76	9	34.61	48.065	51.91	Tibeto-Burman
14	Jharkhand (JH)	63	Gorait	63	31	49.2	27	42.85	5	7.93	67.45	32.53	Austro-Asiatic
15	Uttaranchal (UK)	5	Sah	5	2	40	1	20	2	40	40	60	Dravidian
16	Jammu & Kashmir (JK)	15	Kashmiri Pandits	15	7	46.66	5	33.33	3	20	56.66	43.33	Indo-European
17	Orissa (OD)	85	Kandha	85	41	48.23	20	23.52	24	28.23	47.635	52.345	Indo-European
18	Andaman islands (AI)	39	Andamanese	16	5	31.25	10	62.5	1	6.25	78.125	21.875	Andamanese
			Onge	23	12	52.17	3	13.04	8	34.78	39.125	60.865	Onge
19	Nicobar islands (NI)	23	Nicobarese	23	15	65.21	5	21.73	3	13.04	54.335	45.645	Nicobarese
20	Assam (AS)	27	Nyshi	27	11	40.74	7	25.92	9	33.33	46.29	53.7	Tibeto-Burman
21	Kerala (KE)	61	Kurumban	61	23	37.7	29	47.54	9	14.05	66.39	33.6	Dravidian

I/D Insertion/Deletion, D/D Deletion/Deletion, I/I Insertion/Insertion.

## Discussion

It has been shown initially that the mutations in the *cTnT* gene are responsible for approximately 15% cases of familial hypertrophic cardiomyopathy (FHCM) [27]. However, subsequent studies have identified *cTnT* gene mutations in familial dilated (FDCM) [28], restrictive (RCM) [29], and left ventricular noncompaction [30], cardiomyopathies. Interestingly, our study of *cTnT* gene in 147 dilated cardiomyopathy (DCM) patients against 207 ethnically matched healthy controls had revealed a total of 15 SNPs and a 5 bp INDEL, including a novel heterozygous C→T at nucleotide g.14351 in exon 10 of *cTnT* gene in a DCM patient. The mutation had substituted polar-neutral amino acid tryptophan for a highly conserved wild type basic amino acid arginine within the amino terminal tail at residue 144 (R144W) of *cTnT*.

The R144W mutation was found to be within the tropomyosin-binding domain of *cTnT* and alters the charge of the residue, so it is expected to have a significant impact on the structure and function of the protein. Later, screening of this mutation in all the available members of a large four generations family had revealed the presence of this heterozygous R144W mutation in three affected individuals of the family (Fig. 2), suggesting that it is an autosomal dominant trait. However, evaluation of 207 unrelated healthy control individuals and 162 HCM patients [25] did not show this (R144W) mutation.

The proband and 3 individuals positive for R144W mutation had showed clinical features, that are typical for DCM, specifically, left ventricular dilatation and depressed contractile function (Table 2). The sudden cardiac death (SCD) was also been reported in the family, two individuals, who were diagnosed with dilated cardiomyopathy (DCM), had died due to severe congestive heart failure at the age of 45 and 25 years, these deceased individuals were developed their cardiac condition in the second and third decades of their life, respectively. However, a 66-year-old individual in the same family has started having mild symptoms only at his sixties (Fig. 2). Thus, the age of onset and the severity of the disease are highly variable within the family, suggesting that, in many cases, the scenario is more complex, if the secondary etiological factors, such as lifestyle and environment are involved (Fig. 2).

In addition, this amino acid tail residue arginine at 144 in human *cTnT* is evolutionarily conserved across species, including mouse, rat, chicken, quail, and nematode etc. (Fig. 3). It appears that the amino-terminal tail of *cTnT* is essential for assembly and anchoring of the troponin-tropomyosin complex onto the thin filament [31]–[33]. The troponin-tropomyosin complex is a Ca^2+^ sensitive switch that regulates actin-myosin interaction. The troponin complex (Troponins T, I, and C) is anchored to tropomyosin predominantly by troponin T and to a lesser extent by troponin I, and Troponin C interacts with these two troponins T and I [33]. During systole, Ca^2+^ binds to *cTnC* and initiates conformational changes of the troponin complex that attenuate the inhibitory effect of *cTnI*. Results in the release of active sites of the actin gene and this enables the myosin head of the thick filament to interact with it and generate force. The Ca^2+^ concentration controls *cTnC-cTnT* interaction, which is important for regulating sliding velocity between thick and thin filaments. Interestingly, recent studies have proposed that *cTnT* is critical, not only for the structural integrity of the troponin complex, but also for sarcomere assembly and cardiac contractility [31]–[33].

In general, most of the reported mutations that were responsible for the disease phenotype of dilated (DCM) were in the amino-terminal tail of *cTnT* (exons 10 and 13) [34]–[36]. Moreover, no mutations responsible for familial hypertrophic cardiomyopathies have ever been identified in either of these exons, 10 and 13 [36]. Study of [37] some of the published mutations [(R131W [35] and R141W [36] in exon 10), and (Lys 210 del [34,35], R205L [35] in exon 13)], in the amino-terminal tail of *TNNT2* gene reported to be responsible for dilated cardiomyopathy (DCM); along with other 4 thin filaments mutations, reconstituted with a 1∶1 ratio of mutant∶wild type proteins, all showed reduced Ca^2+^ sensitivity of activation in ATPase and motility assays, and all showed lower maximum Ca^2+^ activation.

Integration of the cTnT mutations (R141W [36] and R205L [35], into skinned guinea pig cardiac trabeculae also reduced Ca^2+^ sensitivity of force generation [37]. Therefore, diverse thin filament DCM mutations appeared to affect different aspects of regulatory function, nevertheless changing contractility in a consistent manner. Further [37]stated that the DCM mutations depressed myofibrillar function, an effect opposite to that of HCM-causing thin filament mutations, and suggested that decreased contractility might trigger pathways that ultimately lead to the clinical phenotype. Generated knock-in mice [38] with a reported mutation, K210-del [34], [35] in exon 13 of *cTnT* gene, and found that cardiac muscle fibers from mutant mice showed significantly lower Ca^2+^ sensitivity in force generation than those from wild type mice [38].

Compound heterozygosity (double and triple mutations) had been reported to cause HCM phenotype [18], [19]. Therefore, we have further analyzed the patient and three of his family members carrying R144W mutation having DCM phenotype with eight other genes (*β-MYH7, MYBPC3, TPM1, TNNI3, TTN, ACTC, MYL2* and *MYL3*), to rule out compound heterozygosity. Our analysis revealed that none of these 4 individuals showed any disease causing mutations in eight of the above-mentioned genes, except with few polymorphic variants. This had further confirmed that the missense mutation R144W in *cTnT* gene is essentially responsible for FDCM phenotype in our study family.

Of 15 SNPs, we have identified a novel splice acceptor site mutation (G→A) at g.16283 in intron 12 (rs) of *cTnT* gene in a 63-year-old male DCM patient (Table 1; Fig. 1B). Unfortunately, we were unable to get the family samples for further analysis. The splice acceptor site variant might create an alternative acceptor site for splicing, which may results in the inclusion or exclusion of amino acid (glutamine) or the complete skipping of the exon (9 nucleotides). As a result, this alternately spliced transcript might form isoforms, which may be expressed in the human heart are expected to be responsible for the disease phenotype; however, this need to be studied further.

Interestingly, we also found a variant C→T at g.15179 in intron11 of *cTnT* gene exclusively in a DCM, was predicted to affect splicing. But we have unable to collect the family samples. We have compared the genotype and allele frequencies of polymorphic SNPs observed in this study with HapMap (NCBI database; www.ncbi.nlm.nih.gov/projects/SNP/snp) populations (HER_ASIAN-PANEL; HER_HISP-PANEL;HER_CEPH-PANEL; HER_YORUB-PANE) (Fig.).

We have compared the 5 bp INDEL frequencies in 147 DCM against 207 healthy controls along with 2092 randomly selected individuals belonging to 39 ethnic and endogamous populations inhabited in 19 states of India (Table 6). Our study revealed that the 5 bp INDEL frequencies were found to be almost same in DCM and the controls; nevertheless this 5 bp INDEL frequency was high in South and the Northwest regions of Indian populations, and HCM [25] (Fig. 6B).

In conclusion, we strongly suggest that the novel unique/private R144W mutation identified in our present study is associated with FDCM. The high level of endogamy in Indian populations along with the influence of evolutionary forces such as genetic drift, fragmentation and long-term isolation, has kept the Indian populations diverse and distant [39]. Hence, the unique mutation observed in this study is not surprising. Our study further suggests that it is important to understand the fundamental genetics (mutation) cause and its impact on disease phenotype, this will certainly lead to adopt novel approaches for the diagnosis and treatment of disease.

## Materials and Methods

### Ethical statement

All of the DNA samples analyzed in the present study were derived from blood samples that were collected with the informed written consent of the donors. The Institutional Ethics Committee of Care Hospitals, Hyderabad, India; and the CSIR-Centre for Cellular and Molecular Biology, Hyderabad, India, have approved the study. This study conforms to the principles outlined in the Declaration of Helsinki (WMA World Medical Association Declaration of Helsinki). The study subjects were all South Indian patients with dilated cardiomyopathy (DCM), diagnosed based on the NYHA (New York Heart Association, 1994), and WHO (www.who.int/cardiovascular_diseases) guidelines.

### Inclusion criteria

Dilated cardiomyopathy (DCM) is characterized by left ventricular enlargement (LVE), and when echocardiography demonstrated a depressed systolic dysfunction with an ejection fraction (LVEF) <45–50% and/or fractional shortening <25%.

### Exclusion criteria

Patients with concomitant disease like; autoimmune disease, cancer, as well as patients with coronary artery disease (CAD), ventricular outflow tract obstructions and with advanced chronic renal failure (CRF), were excluded.

### Genetic analysis

We have sequenced all the exons, including the exon-intron boundaries (5373 bp length) of Troponin T2 (*cTnT*) gene (Table S1), of clinically well-characterized 147 DCM against ethnically matched 207 healthy controls. (Text S1)

### In silico analysis

To evaluate whether the SNPs observed exclusively in DCM have any potential cause for the defect in splicing, we have analyzed these sites with ASD Workbench wrapper (http://www.ebi.ac.uk/asd-srv/wb.cgi) tools, such as poly-pyrimidine tract (PPT), and branch-points (BP). The novel SNPs observed in this study were subjected to identify the presence of PPT and BP binding sites for splicing factors, and exonic splicing enhancers/silencers (ESE/ESS) or intronic splicing enhancers/silencers (ISE/ISS), respectively. Splicing Rainbow tool searches for the SR proteins (serine/arginine-rich) as well as hnRNP motifs.

## Supporting Information

Text S1 **Supporting Materials and Methods.**
(DOCX)

Table S1 **Primers used for the amplification and sequencing of troponin t2 (tnnt2) gene.**
(DOCX)
