# Supplementary material for: A Novel Arginine to Tryptophan (R144W) Mutation in Troponin T (cTnT) Gene in an Indian Multigenerational Family with Dilated Cardiomyopathy (FDCM)
Source: PLoS One. 2014 Jul 3;9(7):e101451. doi: 10.1371/journal.pone.0101451 (PMC4081629; doi:10.1371/journal.pone.0101451)
Supplement: Text S1 — Supporting Materials and Methods. (DOCX) [file pone.0101451.s001.docx]

**Supplementary Text**

**Material And Method Section**

*DNA isolation*

DNA was isolated from blood samples using the standard protocol: Erythrocytes were lysed with 15.0 mL of erythrocyte lysis buffer [containing 10mM Tris at pH 8.0, 320 mM sucrose, 5 mM *MgCl_2_* and 1% Triton X-100; from Sigma Chemical Company, St. Louis, MO, USA] for 5 min. Leucocytes were pelleted by centrifugation at 500g for 5 min and the pellet was dissolved in 8.0 ml of leucocyte lysis buffer [(400 mM Tris, 60 mM EDTA, 150 mM NaCl, and 1% SDS) from Sigma Chemical Company, St. Louis, MO, USA] and was mixed thoroughly. To this lysate, 2.0 ml of 5M sodium perchlorate (E. Merck, Darmstadt, Germany) was added and mixed thoroughly for 2–3 min. DNA was precipitated after extracting once with phenol:chloroform (1:1) and than with chloroform. DNA was washed with 70% ethanol and dissolved in TE (10 mM Tris at pH 8.0 and 1 mM EDTA) buffer.

*Genetic analysis*

Primers covering all the exons, exon-intron boundaries covering 5373 bp of *cTnT* gene, were obtained from <http://genepath.med.harvard.edu>. These primers (Table. S1) were synthesized using an ABI 392 oligo synthesizer (Perkin–Elmer, Foster City, CA, USA), and PCRs (polymerase chain reactions) were carried out under standard conditions, containing 50 ng of genomic DNA as template, 5 pM of both forward and reverse primers, 200 mM dNTPs, 10X PCR buffer having 1.5 mM MgCl_2_ and 1U of Ampli. Taq Gold (Perkin–Elmer). Amplification was carried out in a thermal cycler (MJ Research, Waltham, MA, USA) using the following cycling conditions: 94^o^ C for 5 min, 35 cycles at 94^o^C for 1 min, 55–60^o^C for 1 min, 72^o^ C for 1 min and followed by a final extension at 72^o^ C for 10 min. Amplified PCR products were purified by treating them with ExoSAP-IT (Exonuclease 1 and Shrimp alkaline phosphatase; USB Corporation, 26, 111 Miles Road, Cleveland, Ohio 44128, USA), according to the manufacturer’s instructions. The purified PCR products were bi-directionally sequenced using the ABI Big Dye Terminator cycle sequencing kit (Perkin–Elmer, Foster City, CA, USA) and analyzed using on ABI 3730 DNA Analyzer (Applied Bio-systems, Foster City, CA, USA). Sequences were edited and compared with the reference sequence (*cTnT*) using Auto-Assembler software (Applied Bio-systems, Foster City, CA, USA).
