# Supplementary material for: A Novel Arginine to Tryptophan (R144W) Mutation in Troponin T (cTnT) Gene in an Indian Multigenerational Family with Dilated Cardiomyopathy (FDCM)
Source: PLoS One. 2014 Jul 3;9(7):e101451. doi: 10.1371/journal.pone.0101451 (PMC4081629; doi:10.1371/journal.pone.0101451)
Supplement: Table S1 — Primers used for the amplification and sequencing of troponin t2 (tnnt2) gene. (DOCX) [file pone.0101451.s002.docx]

| **TABLE S1. PRIMERS USED FOR THE AMPLIFICATION AND SEQUENCING OF TROPONIN T2 (*TNNT2*) GENE** | | | | | | |
| --- | --- | --- | --- | --- | --- | --- |
| **S:No** | **Exons** | **Forward Primers** | **Reverse Primers** | **Exons (bp)** | **Ampli size (bp)** | **Ann tp ^0^C** |
| 1 | TNNT2-2 | ACAGCTCATGAGGGGTGGAACTA | GTGCTCTGCCTGGGATCTACAACC | 85 | 376 | 65 |
| 2 | TNNT2-3,4 | ATGAGAACGGCAGGCCAGGCTAGTG | GTTTGCCTCAAGACCCGAGCAACC | 11,15 | 506 | 65 |
| 3 | TNNT2-5 | GTGGCGGGAGGTAGCCGACAGT | TGGGCAATCAATGGTTGAATCTTA | 66 | 403 | 65 |
| 4 | TNNT2-6 | TTGACCCAGCGCTTCTCTTGTGTC | ACTGGGTGCCACCAATGCAACTTC | 36 | 449 | 65 |
| 5 | TNNT2-7 | CCAGTGCCGGGAGGGACTCAC | CAGCCCGTGTCCACTGCACCATAC | 34 | 262 | 65 |
| 6 | TNNT2-8 | GGATCAGGCCCTGCCTGTCCTGACA | TCCTCCTCCTCTTTCTTCCTGTTCT | 61 | 538 | 62 |
| 7 | TNNT2-9 | GCCAGGCCCTGCCAGAGGTCTT | CCCTGGGGGAGGCCTGAAACAG | 117 | 494 | 70 |
| 8 | TNNT2-10 | ACGTCCGTGGAGCTGGTTGAAAGT | CCCGGCCAATATTGTCTCTTGACT | 78 | 373 | 62 |
| 9 | TNNT2-11 | TGGGAGCTACCCTCTCAGAA | CACAGCAGCTGGGAATCTCT | 111 | 369 | 60 |
| 10 | TNNT2-12 | GTAAACCCGGCTGACTACAG | AGCCAGCCCAATCTCTTCAC | 9 | 258 | 62 |
| 11 | TNNT2-13 | CAGGGGGTTTGGGGAGGGTTAG | GTGGGGCACCTGCTCAGTTCTCT | 110 | 402 | 60 |
| 12 | TNNT2-14 | GGAGGGCCCTTTCTTACTGGAC | CCGGACCCAGTGAACCAGGAGGAG | 91 | 207 | 68 |
| 13 | TNNT2-15 | GCCCCTCCTGACCCTTAACTATCC | CGGAGGAGCCAGAGAAGGAAACCT | 41 | 353 | 62 |
| 14 | TNNT2-16 | GGGGGTGAAATGTGGGGCGGAGAA | GTGTGGGGGCAGGCAGGAGTGGTG | 46 | 383 | 62 |

Ampli size –Amplicon size, Ann tp- Annealing temperature,
